# “Don’t Get Your Meat Where You Get Your Bread”: Beliefs and Advice about Workplace Romance

**DOI:** 10.3390/bs12080278

**Published:** 2022-08-11

**Authors:** Betty H. La France

**Affiliations:** Department of Communication, Northern Illinois University, DeKalb, IL 60115, USA; blafrance@niu.edu

**Keywords:** workplace romance, sexual interactions, social supportive communication, advice

## Abstract

This investigation identified contemporary beliefs about workplace romance and compared how those beliefs have changed since 1986. Different kinds of advice about workplace romance, and how that advice was related to extant beliefs, were also evaluated. A nationwide sample (*N* = 259) of organizational members with a variety of professional experiences responded to an anonymous online survey. Results indicated that there were three fundamental underlying beliefs about workplace romance: workplace romance is valuable, the right to demand privacy about workplace romance, and anti-workplace romance. Different types of advice—encouraging, warning, gender concern, and silence—were related to these existing beliefs. The substantial associations between beliefs and advice provide evidence for an implicit theory of workplace romance. Personal experience with such relationships was strongly related to the belief that workplace romance is valuable and the right to demand privacy about workplace romance. Additionally, personal experience was also associated with providing advice promoting workplace romance and advocating that employees should remain silent about engaging in such relationships. These results are discussed within the theoretical lens of boundary blending between the work sphere and the private sphere of social life.

## 1. Introduction

On 2 February 2022, *The New York Times* reported that Jeff Zucker had resigned his position as the president of CNN as the result of a consensual romantic relationship with a subordinate [1]. This workplace romance, it was reported, was with Allison Gollust, who was CNN’s executive vice president. According to Zucker and Gollust, their close friendship and professional relationship had been maintained for 20 years, but it had evolved during COVID-19 to include a romantic relationship. Neither Zucker nor Gollust had reported the relationship to CNN, which is a violation of its organizational policy. The *Times* went on to note that Zucker and Gollust’s relationship had surfaced during an internal investigation of Chris Cuomo, a CNN anchor who was fired because of his political entanglements with his brother, former New York Governor Andrew M. Cuomo. The governor had been accused of sexual misconduct.

This example succinctly highlights different aspects of sexuality within the workplace. Sexual harassment has received considerable cultural, legal, and academic attention, but, increasingly, scholarly research into consensual romantic relationships that develop within organizations has increased [2,3,4,5,6,7,8,9,10,11,12]. In part, this interest in workplace romance has developed because of the blurring of tangible and psychological professional and personal spaces. The current investigation adds to the burgeoning literature by examining the blending of spaces through determining how beliefs about workplace romance have changed over the previous four decades. It is also argued that these sets of beliefs form an implicit theory about workplace romance, including how we communicate about them, especially the advice given about engaging in such relationships. Consequently, social supportive messages that provide advice about workplace romance are assessed, and how contemporary beliefs are associated with that advice are explored. To begin, a brief discussion of how workplace romance is conceptualized, and what research has revealed about important aspects of these kinds of relationships is presented next.

### 1.1. Workplace Romance

In a recent report examining organizational behavior during COVID-19, 33% of U.S. workers reported having engaged in a workplace romance [13]. Of these relationships, most were with peers (65%), but some organizational members had relationships with their superiors (19%) or subordinates (12%). The report also noted that the number of people who said they were participating in or had participated in a workplace romance had increased 6% since before COVID-19. Furthermore, half of the workers reported having a crush on a coworker, and 75% of organizational members were comfortable with people in the workplace engaging in romantic relationships. These numbers demonstrate the popularity of romantic relationships at work.

Horan and Chory defined workplace romance as, “a nonplatonic relationship between two members of an organization in which sexual attraction is present, affection is communicated, and both members recognize the relationship to be something more than just professional and platonic” [6] (p. 565). This conceptualization is, in part, based on how workplace romance has been examined and the language and relational descriptors used in previous research. Historically, terms such as love, flings, organizational romance, close and personal relationships, sexual behavior at work, sincere affection [7], intimate relationships, romantic relationships [8], sexual relationships, dating [6], hook ups [9], extramarital affairs, friends with benefits [10], workplace intimacy, sexual intimacy in the workplace [11], romantic relationships between organizational members, and sexual romance [12] have been used alternately to describe workplace romance. The usefulness of Horan and Chory’s definition is based on its broad conceptualization that includes these dimensions of workplace romance [6].

Using Clark’s work/family border theory, Horan and Chory argued that workplace romances are sites where private lives are blended into the domain of work [6,14]. Clark’s theoretical explication of the work/family border highlights the importance of what were once considered separate domains during the industrial revolution where work in industry was separate and distinct from life at home [14]. Conceptualizing these public and private experiential spheres as distinctly separate domains of social life has had meaningful interpersonal and cultural consequences [15].

Perhaps the most relevant consequence of separating these spheres of social life includes how workplace romance is perceived. Clark’s theoretical explication of borders provides one useful way of examining workplace romance. Border strength (the tangible and psychological division of appropriate role behaviors), flexibility (the extent to which the border can change), blending (the border territory where distinct role behaviors become less distinct), and permeability (the degree to which elements of one domain may appear in the other domain [14] (pp. 756–757)) are facets of the border between the private/family and professional/work spheres of social life. Blending, Clark argued, specifically occurs when flexibility and permeability are high [14]. A workplace romance, then, is a professional relationship that exhibits flexible and permeable borders where sexual activity, intimacy, and task-related work are blended. Organizational members may struggle with these highly flexible and permeable borders, especially as those employees form, maintain, and change their beliefs about coworkers engaging in workplace romances.

Despite the popularity of workplace romances, actual and desired, negative beliefs about such interactions are also common [5]. This apparent paradox represents organizational members’ ambivalence about these kinds of relationships. Therefore, organizational members’ beliefs about romance in the professional sphere are discussed next.

### 1.2. Beliefs about Workplace Romance

Almost 40 years ago, Powell asked what “tomorrow’s managers” thought about sexual intimacy in the workplace [11] (p. 30). These future managers were undergraduate students in an introductory management course and part-time graduate students taking MBA courses. Powell asked his participants to respond to statements about intimacy in the workplace he had culled from popular media sources and a training handbook for human relations personnel. At that time, the strongest beliefs were that organizational members’ personal lives were not the business of management and supervisors who showed subordinates sexual attention should be reprimanded. The students with more work experience (i.e., MBA students) most stridently agreed with the belief that they would never engage in a workplace romance with a coworker or superior.

The intervening years since Powell’s work have likely resulted in a change in perceptions about workplace romance. Indeed, Chory and Gillen Hoke found that millennials (individuals born between 1981 and 1996) perceive and react to workplace romance generally positively [9,16]. Additionally, millennials reported experiences consistent with a permeability of the work sphere and the private sphere of social life. Thus, an assessment of how specific beliefs have changed over time would be useful in understanding contemporary perspectives of workplace romances. As such, Powell’s assessment of such beliefs serves as a specific reference point to examine how beliefs have shifted over the decades. Thus, the first research questions are:

RQ1: what are the contemporary beliefs about workplace romance?

RQ2: to what extent have beliefs about workplace romance changed over time?

Pierce’s work also examined attitudes toward sexual intimacy at work [12]. For Pierce, attitudes regarding workplace romance focused on beliefs about sexual interactions between organizational members. He predicted and found that men held more positive beliefs about such relationships than women. Pierce argued that this result reflected the higher social and tangible organizational costs that existed for women compared with men. Pierce’s results were also consistent with Powell’s work that demonstrated female undergraduate students and MBA graduate students held more negative beliefs about workplace romance than men [11]. In addition to the costs of such relationships, Powell argued that women more than men were likely to perceive and be concerned about sexual harassment.

Chory and Gillen Hoke found that millennial men, more than women, reported no negative effects of workplace romance [9]. These men also identified substantially more positive organizational effects of workplace romance (e.g., working harder) than women [9] (p. 592). Given these reported differences in men’s and women’s perspectives, it is hypothesized that:

**Hypothesis** **1** **(H1):** *women will hold less positive beliefs about workplace romance than men*.

In addition to gender (the term gender (not sex) is used here to note differences between men and women. Gender is used to eliminate the confusion with sex given the context) differences, perceptions of workplace romance differ based on whether the relationship consists of a status difference/hierarchical workplace romance. In this kind of dyad, one party is the superior and the other party is the subordinate. Using scenarios that presented narratives in which heterosexual workplace romances occurred between peers or superior/subordinates, Horan and Chory found that supervisors engaging in such relationships were perceived to be more untrustworthy, less honest, more interpersonally distant, and were more likely to be the targets of deceptive messages from wary organizational members [5]. By comparison, Chan-Serafin et al., found that organizational members who were subordinates in workplace romances suffered considerable organizational costs [3]. Namely, they were less likely to be promoted and less likely to be invited to participate in a development/training activity. Thus, it is predicted that:

**Hypothesis** **2** **(H2):** *beliefs about workplace romance will be more negative for hierarchical workplace romances compared with same-status relationships*.

Dillard, Hale, and Segrin argued that the foundational beliefs that organizational members hold about the nature of workplace romance form a networked system of beliefs that can best be conceptualized as an implicit theory [4]. This theory of beliefs, they argue, includes some beliefs about workplace romance that are not plausible. For example, the advice “all such relationships are bad” views romantic work relationships as unvarying and presumedly as always suffering negative consequences [4] (p. 228). However, another important implication of an implicit theory of workplace romance is that communication about such relationships is driven by the underlying beliefs that employees have about workplace romance.

That communication is one manifestation of underlying cognitions in interpersonal sexual relationships is a common idea [17]. Yet, perhaps the precariousness nature of this link between beliefs and communication in the context of workplace romance is best understood as a manifestation of permeable and flexible boundaries between public and private spheres of social life. Indeed, Powell and Foley assert that romantic workplace relationships are fundamentally different than other organizational relationships because of the strong social norms against such relationships, organizational policies that prohibit such relationships, and workplace romance invites other organizational members to engage in negative judgements about productivity, performance, decision making, and inequities generated, in part, from sexual favoritism [18]. This opportunity for negative evaluation resulting from workplace romance provides employees engaging in such relationships with a kind of social predicament [19]. In addition to accounts and apologies as acts of remediation for private/public border blending, seeking or providing social support in the form of advice may serve as a communication strategy for managing that border. How advice offered about workplace romance might be related to extant beliefs within an implicit theory of workplace romance is an interesting empirical question. Thus, previous research investigating advice is discussed next.

### 1.3. Advice about Workplace Romance

Offering help is a fundamental dimension of interpersonal friendships [20]. One way to help someone is to provide social support [21]. Messages that contain advice are best understood as a kind of supportive communication [22,23,24], and advice is defined as a “recommendation about what to do, think, or feel to manage a situation” [25] (p. 913).

Advice messages are necessarily complicated. They often are construed as threatening in that they likely outwardly acknowledge that the recipient of advice has a problematic situation about which they are incompetent at addressing, and the activity of providing advice serves to decrease the recipient’s autonomy in addressing that situation [23,26]. Advice also has the potential to be perceived as unhelpful because it can be critical, minimizing, or pessimistic [27]. Additionally, there is a difference in outcomes of advice depending on whether that advice was or was not solicited [28,29]. Investigations of advice have examined these and related complications to determine how advice is perceived.

What constitutes good advice is dependent on a wide variety of factors. Typically, advice has been assessed in terms of outcomes. That is, recipients of advice messages think that they have received high quality advice to the extent to which those messages are helpful, effective, and supportive [30]. Furthermore, explicit advice is more effective than indirect or vague advice [24]. However, there has been little empirical work to date about how specific workplace romance advice is evaluated. What kinds of advice people will perceive as good advice regarding workplace romance is an important empirical question. Thus, the following research question was asked:

RQ3: how is advice about workplace romance evaluated?

Evaluations of advice messages are also influenced by how those messages conform to extant cognitions. MacGeorge et al., found that advice that contained content that confirmed recipients’ existing cognitions (i.e., plans to act) led to a host of positive assessments, including that the advice was polite, high quality, likely to be implemented, and recipients reported they were satisfied with the conversation with an advice giver who they found helpful [30]. Similarly, advice content that was consistent with confirming cognitions rated the advice giver as more trustworthy, similar, and likable [31]. The confirming content of advice messages suggests that, although there are a host of variables that people use to judge advice messages, such as context, timing, advisor qualities, etc., advice that is consistent with underlying beliefs is perceived positively. This finding is consistent with a sort of confirmation bias [32]. Thus, it is hypothesized that:

**Hypothesis** **3** **(H3):** *perceptions about advice about workplace romance will be related to extant beliefs about such relationships*.

## 2. Materials and Methods

### 2.1. Participants and Procedure

The sample (*N* = 259) included persons who identified as women (62%), men (36%), and nonbinary (2%). Individuals reported that they were heterosexual (85%), bisexual (10%), gay or lesbian (3%), or not listed (2%). Participants indicated that they were White (78%), Asian (7%), Black (5%), Hispanic (5%), or Mixed (4%) ethnicity (1% not listed), and they were, on average, 36 years old (*M* = 35.94, *Mdn* = 34.00, *Mo* = 28.00, *SD* = 10.85, *Min*. = 19, *Max*. = 73).

An anonymous online questionnaire was distributed via snowball sampling and using Prolific Academic, a high-quality online data collection platform (statistical analyses compering these two samples revealed a few significant differences. Mainly, the participants in the Prolific sample (*N* = 197) tended to be younger and had fewer years of organizational experience than the snowball sample participants (*N* = 62). All statistical differences in these samples, however, were accounted for in the statistical analyses reported here). For the snowball sample, an email that included a link to the online questionnaire was sent to known organizational employees (outside academia). Recipients were encouraged to complete the online questionnaire and forward the link or post it on social media. All participants met three inclusion criteria: they were at least 18 years old, they had to be employed either part-time or full-time during the previous three years, and they had at least one romantic sexual relationship with anyone in their lifetime. Participants were informed that the online questionnaire included sensitive questions about romantic sexual relationships within the workplace, and they were instructed to use a personal device during their private time to complete the online questionnaire. The online questionnaire contained the following items measuring the constructs of interest and those items were randomly ordered. Demographic information was also collected. A quality control item was included to ensure adequate attention and participation.

### 2.2. Measures

Participants first responded to a series of questions about their professional work experiences. These experiences included how many years worked, organizational position, reporting behavior, etc., and participants were asked about their personal experience with workplace romance. Additionally, participants responded to whether their organization had a sexual harassment policy, a policy about members dating one another, and whether members thought organizations should have policies about its members engaging in consensual sexual activities with each other.

#### 2.2.1. Workplace Romance

Fifteen items from Powell’s 18-item Beliefs Concerning Sexual Intimacy in the Workplace scale were used to measure beliefs about workplace romance [11] (see Table 1). Three items referring to sexual orientation (e.g., whether or not a person is homosexual should be considered before he or she is appointed to a managerial job) were not included in the current investigation. Participants responded to each item using a 7-point Likert-type scale, where 1 = strongly disagree and 7 = strongly agree.

Although no factor analytic results were reported, Powell argued that the items on his scale represented five dimensions of beliefs: value of sexual intimacy, sexual habits and personal life as a concern of management, desirability of managerial actions to discourage sexual intimacy, sexually oriented behavior engaged in, and acceptability of sexual orientation [11] (p. 32). To determine statistically whether there was an underlying factor structure for this scale, the 15 items to which participants responded in the current study were subjected to an exploratory factor analysis. It was anticipated that some factors would likely be correlated, so promax (oblique) rotation and principal axis factors were used. Items were retained if: the inter-item correlations were positive, inspection of the residual matrices of the predicted and observed inter-item correlations displayed small residuals, factor loadings ≥0.40, eigen values for each factor were >1.00, no item cross-loaded on another factor ≥0.40, two or more items loaded on one factor, and reliability estimates were acceptable. This analysis resulted in three factors, and items within each subscale were averaged: workplace romance is valuable (items 1, 2, 3, 11; *M* = 3.46, *SD* = 1.04, α = 0.73), the right to demand privacy about workplace romance (items 4, 5, 9; *M* = 5.26, *SD* = 1.04, α = 0.53), and anti-workplace romance (items 14, 15; *M* = 4.49, *SD* = 1.68, α = 0.73).

#### 2.2.2. Advice about Workplace Romance

A 26-item scale assessing advice messages was specifically created for the current study. Items were generated by examining lay advice offered in popular culture artifacts including magazines [33,34,35,36], etc., (see Table 2). Participants were prompted to consider advice they would offer a friend about engaging in consensual sexual activities with someone they work with. They responded to each statement using the following 4-point scale, 1 = worst advice, 2 = bad advice, 3 = good advice, 4 = great advice. All 26 items were subjected to an exploratory factor analysis with promax (oblique) rotation and principal axis factors. Applying the same criteria as noted above, four factors were created by averaging items within each subscale: encouraging (items 5, 8, 13, 16, 17, 19, 21, 25; *M* = 2.28, *SD* = 0.49, α = 0.82), warning (3, 6, 9, 10, 14, 15, 20, 23, 26; *M* = 3.02, *SD* = 0.44, α = 0.80), gender concern (11, 12; *M* = 2.18, *SD* = 0.72, α = 0.86), and silence (1, 2; *M* = 2.42, *SD* = 0.65, α = 0.77). An open-ended question asked respondents to report any other advice they had heard about engaging in sexual activities with someone with whom they worked.

## 3. Results

### 3.1. Answering the Research Questions and Testing the Hypotheses

The first research question (RQ1) asked: what are the current beliefs about workplace romance? Table 1 presents the item means and standard deviations for organizational members’ beliefs about these types of relationships. The most endorsed beliefs were: supervisors who direct sexual attention toward their subordinates should be reprimanded, a person’s personal life is not the business of management, I would never get intimately involved with my supervisor, and a manager should be unconcerned with an employee’s sexual habits. Organizational members did not endorse that: sexual relations foster better communication between the workers involved, I would go along with sexually oriented behavior that was common in my work group, and some sexual intimacy among coworkers can create a more harmonious work environment.

Examining the correlations (see Table 3) between the three kinds of beliefs revealed a significant positive correlation between workplace romance is valuable and the right to demand privacy about workplace romance, *r* = 0.28, *p* < 0.001. This finding indicates that beliefs expressing the idea that workplace romances are good for professional life were associated with beliefs that organizational members’ personal lives should remain private and should not be controlled by management. Anti-workplace romance beliefs were negatively correlated with workplace romance is valuable beliefs, *r* = −0.45, *p* < 0.001, and anti-workplace romance beliefs were also negatively correlated with organizational members’ right to demand privacy about workplace romance, *r* = −0.29, *p* < 0.001. This pattern indicates that organizational members who did not believe they would personally engage in workplace romances were less likely to see such relationships within the workplace as valuable and were less likely to demand the right to privacy about such relationships.

The second research question (RQ2) asked about the extent to which beliefs about workplace romance have changed over time (i.e., 1986 and 2022). Table 1 also contains the means that Powell reported [11]. A series of independent samples *t*-tests were performed comparing all 15 items. Powell did not report standard deviations, therefore an average standard deviation was calculated from the standard deviations in the current sample. That average was used to calculate the mean difference for each of Powell’s items and the belief items for the current sample. Results revealed that over time, three beliefs had changed such that participants more strongly endorsed those beliefs: sexual relations foster better communication between the workers involved, I would never get intimately involved with my supervisor, and when two workers cultivate a relationship that eventually leads to marriage it enhances their creativity as a unit and helps their company’s bottom line. However, four beliefs had changed such that participants were more reluctant to endorse those beliefs: a certain amount of sexual joking and innuendo in the workplace should be tolerated, management should take strong steps to discourage sexual propositions toward coworkers, it is alright for someone to dress attractively to draw the attention of coworkers, and I would be offended by a coworker flirting with the supervisor.

Hypothesis 1 (H1) predicted that women would hold less positive beliefs than men about workplace romances. Independent samples *t*-tests were conducted on the 15 items that measured workplace romance. Results revealed that only two beliefs displayed a gender difference: “Any worker who directs sexual attention toward another should be reprimanded”, *M_women_* = 3.93, *SD* = 1.49, *M_men_* = 3.53, *SD* = 1.60, *t*(250) = 1.99, *p* = 0.048, *d* = 0.26, 95% *CI* [0.003, 0.518]; “A certain amount of sexual joking and innuendo in the workplace should be tolerated”, *M_women_* = 3.35, *SD* = 1.59, *M_men_* = 3.83, *SD* = 1.57, *t*(251) = −2.31, *p* = 0.021, *d* = −0.30, 95% *CI* [−0.560, −0.045]. No other significant differences were found. Small effect, *d* = 0.20, power = 0.80 [37]. Women, compared with men, did not believe that sexual attention, joking, or innuendos had a place in the organization.

Hypothesis 2 (H2) anticipated that beliefs in hierarchical workplace romances would be perceived more negatively than peer workplace romances. This hypothesis was tested using a paired samples *t*-test between beliefs about getting intimately involved with a coworker versus having a sexual relationship with a supervisor. Results demonstrated a significant difference between beliefs such that a workplace romance with a coworker was less disagreeable (*M* = 3.64, *SD* = 1.97) compared with a workplace romance with a supervisor (*M* = 5.31, *SD* = 1.81), *t*(257) = −15.39, *p* = 0.000, *d* = −0.96, 95% *CI* [−1.11, −0.81].

Research question three (RQ3) asked about perceptions of advice offered. Table 2 presents what advice organizational members perceived was good advice about engaging in a workplace romance. The highest rated advice was for organizational members to check whether the organization has a policy against romantic relationships at work (one-sample *t*-test, test value = 3), *t*(258) = 18.72, *p* = 0.000, *d* = 1.16, 95% *CI* [1.00, 1.32]. Other advice that organizational members endorsed as good advice was: never date someone who reports to you, *t*(257) = 11.20, *p* = 0.000, *d* = 0.70, 95% *CI* [0.56, 0.83], never date the boss, *t*(258) = 8.57, *p* = 0.000, *d* = 0.53, 95% *CI* [0.40, 0.66], and workplace romances that go wrong make coworkers miserable, *t*(258) = 7.78, *p* = 0.000, *d* = 0.48, 95% *CI* [0.35, 0.61]. The worst advice was: you will get preferential treatment at work if you are dating someone you work with, (one-sample *t*-test, test value = 2), *t*(256) = −6.45, *p* = 0.000, *d* = −0.40, 95% *CI* [−0.53, −0.28]. Other bad advice included: workplace romances help the organization run better, *t*(259) = −5.54, *p* = 0.000, *d* = −0.35, 95% *CI* [−0.47, −0.22], and becoming romantic with a coworker helps complete boring tasks, *t*(259) = −3.13, *p* = 0.002, *d* = −0.20, 95% *CI* [−0.32, −0.07].

A correlational analysis was conducted to determine whether there was any relationship between the four kinds of advice: encouraging, warning, gender concern, and silence (Table 3). Advice that encouraged workplace romances was negatively correlated with advice that warned against such relationships, *r* = −0.46, *p* <0.001, and advice that contained specific gender concern, *r* = −0.27, *p* < 0.001. Encouraging advice was positively correlated with remaining silent about engaging in a workplace romance, *r* = 0.43, *p* < 0.001. Advice that warned against workplace romance was positively correlated with gender concern, *r* = 0.46, *p* < 0.001, but warning advice was negatively correlated with providing advice that suggested organizational members should remain silent about engaging in a workplace romance, *r* = −0.12, *p* = 0.031. Advice that demonstrated concern about gender was negatively correlated with advocating silence about workplace romances, *r* = −0.15, *p* = 0.009.

The final hypothesis (H3) proffered that advice about workplace romance would be related to beliefs about such relationships. Table 3 shows the correlations between beliefs about workplace romance and the advice they would provide about these kinds of relationships. The belief that workplace romance is valuable was positively correlated with advice that encouraged such relationships and with advice messages that instructed organizational members not to discuss their workplace romances (i.e., silence). Participants who demand privacy about workplace romance gave advice that encouraged workplace romance and were unlikely to use messages that warned against such relationships. Individuals who were anti-workplace romance did not encourage others to engage in such relationships, warned bad things would happen if a workplace romance occurred, had specific gender concerns about romantic relationships in the workplace, and were reluctant to encourage organizational members to remain silent about being in a workplace romance.

### 3.2. Organizational Experience

Research participants had a wide variety of organizational experience. During their professional life, they had worked, on average, for 17 years (*M* = 16.82, *SD* = 10.51) with 5 years (*M* = 5.10, *SD* = 5.52) spent in their current position. Most participants reported that they had worked for several employers (3 employers, 15%; 4 employers, 14%; 5 employers 14%; and 10 or more employers, 20%). Organizational members worked primarily in non-management positions (56%) followed by management (32%), top management (7%), and other/not listed positions (5%). Most participants (61%) did not have anyone in their respective organizations report to them, but some participants (39%) noted that some members within their organization did report to them. A vast majority of participants (83%) indicated that their organization had sexual harassment policies, some were unsure if such policies existed (10%), and a few people (7%) reported that their organization had no sexual harassment policies. Almost half of the sample (48%) worked in organizations that did not have policies about members dating each other, other participants (31%) were unsure if any such policies existed within their organization, and some participants (21%) worked in organizations that had organizational policies about dating. Participants were split (yes, 42%; no, 40%; unsure, 18%) regarding whether organizations should have policies about its members engaging in consensual sexual activities with each other.

Research participants also had personal involvements with workplace romance. Organizational members (42%) reported dating someone at work, engaging in sexual activity with another employee (47%), and flirting with someone with whom they work (71%). Other participants reported no such experience with dating another employee (58%), engaging in sexual activity with another organizational member (53%), or flirting with someone within their organization (29%).

As might be expected, age was positively correlated with total years worked, *r* = 0.93, *p* < 0.001, number of years worked in current position, *r* = 0.51, *p* < 0.001, and number of employers, *r* = 0.27, *p* < 0.001. Participants who had worked longer in their professional lifetime had more years at their current position, *r* = 0.51, *p* < 0.001, and had experienced more total employers, *r* = 0.34, *p* < 0.001.

Several analyses were conducted to assess how organizational experience was related to beliefs and advice about workplace romance. Table 4 shows the result of a series of independent samples *t*-tests examining the differences in beliefs and advice about workplace romance based on respondents’ personal experience with those kinds of relationships. The general pattern of findings is clear. People who have dated, flirted, or (especially) engaged in a workplace romance believed that workplace romance is valuable, demanded privacy about such relationships, and were reluctant to endorse anti-workplace romance beliefs. Furthermore, these organizational members with personal experience also expressed advice that encouraged workplace romances, were reluctant to warn against these types of relationships, and endorsed the instruction to remain silent about engaging in a workplace romance.

Table 5 presents the results from a series of one-way ANOVAs with Bonferroni post hoc tests performed to determine the degree to which organizational policy was related to beliefs and perceptions of advice about workplace romance (medium effect, *f* = 0.20, power = 0.80). Participants in organizations with no dating policies believed workplace romance was valuable and demanded privacy about workplace romance. Participants who were in organizations with policies about dating were anti-workplace romance and provided advice that warned against engaging in such relationships. The strongest pattern of effects, however, was for individuals’ perceptions regarding whether organizations should have policies regarding consensual sexual activities between its members. Participants who believed that workplace romance was valuable, demanded privacy about workplace romance, and were not anti-workplace romance, reported that organizations should not have policies about consensual sexual activities between members. These participants also provided advice that encouraged workplace romance and suggested to remain silent if members found themselves involved in these relationships. Organizational members who thought that consensual dating policies should exist were anti-workplace romance and conveyed advice that warned against engaging in workplace romance in addition to offering gender concern for people engaging in workplace romance. Giving advice to remain silent about workplace romance was most likely to be assessed as good advice when organizational members were unsure whether their workplace had any sexual harassment policies.

Independent sample *t*-tests were used to determine whether reporting status influenced beliefs or advice about workplace romance. No differences in beliefs or assessments of advice were found for organizational members based on whether someone in the organization reported to them. A one-way ANOVA was used to assess whether a participant’s managerial role was associated with beliefs and advice about sexual relationships between organizational members. No differences were found.

Age was positively correlated with the advice warning against workplace romance, *r* = 0.14, *p* = 0.013, and expressing gender concern, *r* = 0.14, *p* = 0.012. Similarly, the number of years worked was positively correlated with warning advice, *r* = 0.14, *p* = 0.013, and gender concern, *r* = 0.12, *p* = 0.028. The number of years worked, however, was negatively correlated with anti-workplace romance, *r* = −0.12, *p* = 0.030. Years in an organizational members’ current position was positively correlated with workplace romance as valuable, *r* = 0.11, *p* = 0.042.

### 3.3. Qualitative Complementarity

Most participants (63%) provided responses to the open-ended prompt asking about other advice. Examining these comments revealed that they reflected the types of advice evaluated by the quantitative data, and those comments also provided further support of the advice people heard about workplace romance.

Advice that provided a warning for engaging in workplace romances overwhelmingly appeared in participants’ responses. Organizational members offered the common idiom “Don’t s*** where you eat” and less common idioms such as “Don’t get your meat where you get your bread” and “Don’t dip your pen in the company ink” were provided when thinking about advice concerning workplace romance. These idioms, in part, reflect the implicit theory organizational members have about such relationships.

These qualitative warnings also highlighted the particular problems with hierarchical workplace romances “Never with the boss” and “Never date a boss/subordinate. If you start a relationship with a peer, one of you should probably find another job”. Participants were explicit about acknowledging the power differential in status-difference sexual relationships.

## 4. Discussion

Conceptualizing workplace romances as sites of blending the professional and private spheres of social life [6,14], the current investigation identified contemporary beliefs about those relationships and assessed how beliefs about workplace romance have changed since the 1980s [11]. Advice about workplace romance was related to extant beliefs, and organizational experiences shaped beliefs and were associated with the kind of advice people identified as good advice.

### 4.1. Workplace Romance

The first research question asked: what are the contemporary beliefs about workplace romance? Three foundational beliefs were found: workplace romance is valuable, the right to demand privacy about workplace romance, and anti-workplace romance beliefs. The most steadfast belief that these participants reported was the idea of privacy. Therefore, although there may be legal, historical, and policy-driven expectations that organizational members give up specific privacy rights within their respective organizations, employees’ own belief systems are not consistent with that expectation.

The types of beliefs that organizational members had about workplace romances—workplace romance is valuable, the right to demand privacy about workplace romance, and anti-workplace romance—were substantially related. This finding is consistent with the idea that beliefs about workplace romances are interrelated, which provides further evidence for an implicit theory guiding how people think and communicate about private relationships at work [4]. From a practical perspective, any organizational intervention directed at managing consensual intimate relationships between its employees would need to thoughtfully consider employees’ underlying belief sets to gauge the likelihood that specific recommendations or policies would be followed. Organizations may benefit from moving away from abstinence-only and punishing models that attempt to regulate sexual relationships between organizational members and instead focus on providing training and education regarding how workplace romance and task work may productively and equitably coexist. This idea manifests in a “love contract”, which refers to “a document affirming that a workplace romantic relationship is consensual, that employees involved will not engage in favoritism, and that neither will take any legal action against the employer or each other if the relationship ends” [38] (p. 29).

The second research question asked whether workplace romance beliefs had changed in four decades [11]. Over time, there has been some shifting in how people perceive these kinds of relationships in the workplace. On the one hand, organizational members have become somewhat more romantic about the idea of how workplace romance may improve organizational life. Although they may not believe that overtly sexual behavior is positive, they do think that if marriage results from a workplace romance, then subtle relationship behavior in the organization is justified. This finding is consistent with a contemporary understanding of how permeable boundaries can be beneficial for organizational members [9]. By comparison, there is a greater recognition of the particular hazards of becoming intimately involved with a supervisor.

Individuals who believed that workplace romances were valuable to the organization also believed that employees had a right to demand relational privacy and could imagine themselves as engaging in a workplace romance. Organizational members who were anti-workplace romance in that they believed they could never become intimately involved with someone at work, thought that people within an organization have little right to demand privacy. These findings reflect the dimensions of border blending to which Clark refers in her model of work/family border family [14]. There is a strong sense that people, even when part of an organization, should not be told who they can and cannot see romantically or with whom they can and cannot engage in sexual activity. Indeed, participants were evenly split regarding whether they thought an organization should have any policy regarding sexual activity between its members, and the idea that management should intervene in such relationships was a belief that found decreasing support over time. Overall, organizational members believe in their right to privacy and think workplace romances make professional life more enjoyable.

The first hypothesis (H1) predicted that women would have less positive beliefs about workplace romance than men. This prediction was accurate for only two beliefs. Women, more than men, believed that organizational members should be reprimanded if they directed any sexual attention towards another coworker, and women (compared with men) did not believe that sexual joking and innuendos should be tolerated in the workplace. Although past research has demonstrated that women generally hold more negative beliefs about sexual behavior between organizational employees [9,11,12], the current data demonstrate that there are few gender differences in perceptions of beliefs about workplace romance. Perhaps one reason for this change can be attributed to the MeToo movement, where men have been challenged to reimagine their own beliefs and behaviors about sexually oriented behavior, especially in professional environments [39].

There is significant discrimination in the beliefs about workplace relationships. That is, status-difference/hierarchical workplace romances within organizations are seen as especially detrimental. Consistent with past research [3,5], the second hypothesis (H2) predicted that supervisor/subordinate workplace romances would be viewed particularly negatively. In addition to the quantitative support for this idea, participants’ qualitative responses were pointed: “It is ok if they are not your superior/if they are the same ‘level’ as you”, “Do not date your superior”, and “Only date people on your same level”. It is clear that organizational members do not believe that a workplace romance with a boss is a good idea.

Cognitions provide the foundation for expression [17]. In the case of workplace romance, it was hypothesized (H3) that beliefs about such relationships would be related to advice offered about workplace romance. In part, advice is perceived to be good advice if it confirms existing cognitions [30,31]. Indeed, the advice evaluated in this investigation was significantly related to the types of beliefs organizational members held about workplace romances. Overwhelmingly, employees who perceived that workplace romance was valuable to professional life also thought advice encouraging workplace romance was good advice. These optimistic individuals also refrained from warning against engaging in workplace romances and did not offer gender-concern advice. Perhaps most revelatory, however, was the advice to organizational members who were in workplace romances to keep those entanglements quiet. It could be the case that organizational members perceived that keeping quiet meant that they should avoid overt displays of affection in the workplace, but silence also explicitly recognizes that no one in the organization (e.g., supervisor or human relations department) should be informed that a workplace romance has formed. For these participants, their romantic relationships with coworkers were not the business of management and silence does indeed seem golden.

### 4.2. Organizational Experience

Many organizational members reported dating or engaging in sexual activity with someone at work. Many more participants—in fact, a vast majority of employees—flirted with coworkers. These results are consistent with other reports [13,38]. Furthermore, having personal experience with such workplace romances influenced both beliefs and advice about these relationships. Experience was associated with greater beliefs that workplace romance is valuable and the right to demand privacy about workplace romance. Individuals who had sex with a coworker rated encouraging and silencing advice as good advice. As expected, these same individuals did not offer advice warning of such relationships or demonstrating concern for men’s or women’s particular costs associated with engaging in workplace romances.

### 4.3. Limitation and Conclusions

Although the current investigation was focused on using a baseline to determine how beliefs have changed over time, it is likely the case that a new set of beliefs regarding workplace romance has formed in the last 40 years. For example, specific questions about beliefs regarding public displays of affection, favoritism, time management, and equity likely exist as part of people’s current implicit theory of workplace romance, so future research would benefit from asking questions about those beliefs.

Relatedly, Powell’s original scale did address gay and lesbian relationships in the workplace (e.g., whether or not a person is homosexual should be considered before he or she is appointed to a managerial job) [11] (p. 32). These questions were objectionable and undoubtedly would have angered research participants had they been included. However, the specific challenges that gay men and lesbians experience as organizational members engaging in workplace romances would likely be unique. Indeed, Horan and Chory found that subordinates in same-sex workplace romances with superiors were perceived as less competent than their heterosexual counterparts [40]. Future research would benefit from exploring this issue in a way that reflects contemporary theorizing and operationalizing beliefs and advice that specifically address being gay or lesbian and having a workplace romance.

Because Powell did not report standard deviations with his means for workplace romance beliefs, an average standard deviation was calculated from the standard deviations in the current sample. That average was used to calculate the mean difference for each of Powell’s items and those same items asked in the current survey. This decision may have served to artificially reduce any variation in the means, which would lead to conservative estimates of change over time.

The data reported in the current investigation are driven largely by participants who identified as White. This limitation reflects that further work needs to be done to purposively sample research participants who provide a greater representative perspective regarding workplace romance. It is likely the case that social judgments regarding such relationships vary depending on race and ethnicity.

Overall, this research is important for its identification of beliefs and advice organizational members endorse about workplace romance. It successfully demonstrated how the blending of professional and private spheres of social life represents an organizational reality shaped, in large part, by individuals’ experiences in the organization.

## Figures and Tables

**Table 1:** Workplace romance beliefs.

Item	1986 [11] *M* *N* = 351	2022 *M* *N* = 259	*d*	95% *CI*	*t*	*p*
1. Sexual relations foster better communication between the workers involved. ^a^	2.05 (*SD* = 1.53 *)	3.00 (*SD* = 1.38)	0.65	0.48, 0.81	7.91	<0.01
2. Some sexual intimacy among coworkers can create a more harmonious work environment. ^a^	3.30	3.32 (1.34)	0.01	−0.15, 0.17	1.68	0.867
3. When two workers cultivate a relationship that eventually leads to marriage, it enhances their creativity as a unit and helps their company’s bottom line. ^a^	3.51	3.98 (1.27)	0.33	0.17, 0.49	4.03	<0.01
4. A person’s personal life is not the business of management. ^b^	5.44	5.61 (1.29)	0.12	−0.04, 0.28	1.45	0.148
5. A manager should be unconcerned with an employee’s sexual habits. ^b^	5.14	5.22 (1.58)	0.05	−0.11, 0.21	0.630	0.529
6. Management should take strong steps to discourage sexual propositions toward coworkers.	5.27	4.37 (1.69)	−0.56	−0.73, −0.40	−6.87	<0.01
7. Supervisors who direct sexual attention toward their subordinates should be reprimanded.	5.71	5.76 (1.27)	0.04	−0.13, 0.20	0.428	0.669
8. Any worker who directs sexual attention toward another should be reprimanded.	3.83	3.77 (1.53)	−0.04	−0.20, 0.12	−0.479	0.632
9. It is alright for someone to look for a marriage partner at work. ^b^	5.15	4.95 (1.44)	−0.13	−0.30, 0.03	−1.64	0.102
10. It is alright for someone to dress attractively to draw the attention of coworkers.	4.71	4.17 (1.53)	−0.35	−0.52, −0.19	−4.31	<0.01
11. A certain amount of sexual joking and innuendo in the workplace should be tolerated. ^a^	4.77	3.53 (1.59)	−0.80	−0.97, −0.63	−9.73	<0.01
12. I would be offended by a coworker flirting with the supervisor.	4.42	4.12 (1.70)	−0.19	−0.35, −0.03	−2.28	0.023
13. I would go along with sexually oriented behavior that was common in my work group.	3.10	3.25 (1.52)	0.10	−0.06, 0.26	1.20	0.231
14. I would never get intimately involved with a coworker. ^c^	3.51	3.64 (1.97)	0.08	−0.09, 0.24	0.917	0.359
15. I would never get intimately involved with my supervisor. ^c^	4.43	5.32 (1.81)	0.54	0.38, 0.70	6.57	<0.01

* Estimated SD = 1.53. ^a^ WRV—workplace romance is valuable. ^b^ DPWR—demand privacy about workplace romance. ^c^ AWR—anti-workplace romance. Items without subscripts were not included in the analysis.

**Table 2:** Ranked advice messages about workplace romance.

Item	*M* (*SD*)
6. You better check to see if your organization has a policy against romantic relationships at work. ^b^	3.64 (0.55)
15. Never date someone who reports to you. ^b^	3.47 (0.68)
10. Never date the boss. ^b^	3.40 (0.75)
23. Workplace romances that go wrong make coworkers miserable. ^b^	3.31 (0.64)
17. If you love each other, it is okay to be together. ^a^	3.03 (0.71)
22. You cannot stop people from dating at work.	2.92 (0.65)
26. You can lose your job if you engage in sexual activities with someone at work. ^b^	2.92 (0.76)
21. If you like someone romantically at work, then it is nobody’s business. ^a^	2.91 (0.67)
20. Your coworkers are going to gossip about you. ^b^	2.89 (0.72)
18. It is okay to date someone at work as long as you tell Human Resources.	2.64 (0.83)
9. People will assume any success you have at work is because of who you are dating. ^b^	2.61 (0.81)
3. You will end up hurting your career if you date at work. ^b^	2.55 (0.73)
19. It is natural for people at work who are attracted to each other to engage in sexual activities. ^a^	2.46 (0.77)
1. It is okay to date someone at work as long as you do not talk about it. ^d^	2.46 (0.70)
14. Office romances always go wrong. ^b^	2.40 (0.75)
2. It is okay to engage in sexual activities with a coworker if you keep it quiet. ^d^	2.39 (0.74)
11. Dating a coworker is a problem for women. ^c^	2.21 (0.77)
25. Engaging in sexual activities with a coworker makes sense given how much time you spend together. ^a^	2.15 (0.71)
12. Dating a coworker is a problem for men. ^c^	2.15 (0.76)
7. There is little difference between sexual harassment and flirting at work.	2.10 (0.89)
16. You spend so much time working, it is the only place you can find a romantic partner. ^a^	2.05 (0.73)
24. If you are dating someone at work, then someone else is doing your job for you because you are not focused.	2.02 (0.70)
5. Having a sexual relationship with someone at work makes work fun. ^a^	2.00 (0.76)
13. Becoming romantic with a coworker helps complete boring tasks. ^a^	1.86 (0.69)
8. Workplace romances help the organization run better. ^a^	1.78 (0.65)
4. You will get preferential treatment at work if you are dating someone you work with.	1.75 (0.62)

^a^ Encouraging, ^b^ warning, ^c^ gender concern, ^d^ silence. Items without subscripts were not included in the analysis.

**Table 3:** Correlation between constructs *.

*Construct* Beliefs	WRV	DPWR	AWR	Encouraging	Warning	Gender Concern
WRV						
DPWR	0.28					
AWR	−0.45	−0.29				
**Advice**						
Encouraging	0.68	0.38	−0.55			
Warning	−0.36	−0.23	0.44	−0.46		
Gender Concern	−0.22	−0.24	0.22	−0.27	0.46	
Silence	0.32	0.27	−0.32	0.43	−0.12	−0.15

* *N* = 259. All correlations are significant at, *p* ≤ 0.05. WRV—workplace romance is valuable. DPWR—demand privacy about workplace romance. AWR—anti-workplace romance.

**Table 4:** Difference in workplace romance beliefs and advice based on personal experience.

	Personal Experience		
Beliefs	Dated	Flirted	Sex
	Not Dated	Not Flirted	No Sex
	*d*	*d*	*d*
	95% *CI*	95% *CI*	95% *CI*
WRV	3.72 (0.99)	3.62 (0.99)	3.72 (1.02)
	3.25 (1.01)	3.05 (1.07)	3.22 (1.01)
	0.47	0.57	0.50
	0.22, 0.72 *	0.30, 0.84 *	0.25, 0.74 *
DPWR	5.34 (1.00)	5.31 (1.04)	5.41 (1.00)
	5.18 (1.08)	5.12 (1.03)	5.12 (1.06)
	0.15	0.19	0.28
	−0.10, 0.40	−0.08, 0.45	0.04, 0.53 *
AWR	3.54 (1.36)	4.02 (1.60)	3.57 (1.37)
	5.21 (1.55)	5.63 (1.31)	5.29 (1.52)
	−1.13	−1.05	−1.19
	−1.40, −0.86 *	−1.34, −0.77 *	−1.45, −0.92 *
**Advice**			
Encouraging	2.42 (0.50)	2.37 (0.49)	2.46 (0.50)
	2.17 (0.45)	2.07 (0.45)	2.12 (0.44)
	0.54	0.63	0.71
	0.28, 0.79 *	0.35, 0.90 *	0.46, 0.97 *
Warning	2.92 (0.39)	3.00 (0.42)	2.95 (0.40)
	3.09 (0.46)	3.08 (0.49)	3.08 (0.46)
	−0.38	−0.18	−0.31
	−0.64, −0.13 *	−0.45, 0.09	−0.56, −0.07 *
Gender Concern	2.10 (0.65)	2.15 (0.68)	2.07 (0.63)
	2.24 (0.75)	2.26 (0.80)	2.28 (0.77)
	−0.20	−0.15	−0.29
	−0.45, 0.05	−0.42, 0.12	−0.53, −0.04 *
Silence	2.61 (0.62)	2.55 (0.62)	2.66 (0.61)
	2.29 (0.63)	2.13 (0.62)	2.22 (0.61)
	0.52	0.68	0.72
	0.26, 0.77 *	0.40, 0.95 *	0.46, 0.97 *

**Note**: Dated—dated someone at work. Flirted—flirted with someone at work. Sex—engaged in any kind of sexual activity with someone respondent worked with. WRV—workplace romance is valuable. DPWR—demand privacy about workplace romance. AWR—anti-workplace romance. * *CI* does not contain zero.

**Table 5:** Difference in workplace romance beliefs and advice based on organizational policy.

	Organizational Policy		
Beliefs	Sex Har. Policy	Date Policy	Should Policy
	No Policy	No Policy	No Policy
	Unsure	Unsure	Unsure
	*F*	*F*	*F*
	*ηp* ^2^	*ηp* ^2^	*ηp* ^2^
	*p*	*p*	*p*
WRV	3.40 (1.04)	3.40 _ab_ (1.00)	3.20 _a_ (1.12)
	3.68 (0.47)	3.63 _b_ (1.02)	3.75 _b_ (0.96)
	3.70 (1.24)	3.24 _a_ (1.08)	3.37 _ab_ (0.89)
	1.47	3.55	8.02
	0.01	0.03	0.06
	0.231	0.030	<0.001
DPWR	5.22 (1.05)	4.86 _a_ (1.04)	4.73 _a_ (1.08)
	5.51 (1.14)	5.46 _b_ (0.96)	5.72 _b_ (0.86)
	5.37 (0.84)	5.19 _ab_ (1.07)	5.40 _b_ (0.71)
	0.824	6.70	29.25
	0.01	0.05	0.19
	0.440	0.001	<0.001
AWR	4.53 (1.70)	5.11 _a_ (1.65)	4.91 _a_ (1.74)
	3.88 (1.46)	4.13 _b_ (1.64)	3.98 _b_ (1.68)
	4.59 (1.72)	4.62 _ab_ (1.66)	4.63 _ab_ (1.26)
	1.20	7.01	8.74
	0.01	0.05	0.06
	0.302	0.001	<0.001
**Advice**			
Encouraging	2.26 (0.48)	2.16 (0.51)	2.14 _a_ (0.51)
	2.30 (0.50)	2.35 (0.47)	2.44 _b_ (0.47)
	2.41 (0.57)	2.26 (0.51)	2.24 _a_ (0.41)
	1.19	2.89	10.77
	0.01	0.02	0.08
	0.306	0.058	<0.001
Warning	3.02 (0.44)	3.14 _a_ (0.45)	3.14 _a_ (0.42)
	3.04 (0.52)	2.96 _b_ (0.45)	2.91 _b_ (0.43)
	3.01 (0.41)	3.04 _ab_ (0.40)	2.99 _ab_ (0.44)
	0.026	3.32	8.09
	0.00	0.03	0.06
	0.974	0.038	<0.001
Gender Concern	2.17 (0.73)	2.32 (0.73)	2.36 _a_ (0.77)
	2.44 (0.66)	2.12 (0.71)	2.06 _b_ (0.67)
	2.11 (0.64)	2.20 (0.72)	2.07 _ab_ (0.61)
	1.29	1.52	5.80
	0.01	0.01	0.04
	0.278	0.220	0.003
Silence	2.39 _a_ (0.66)	2.33 (0.65)	2.24 _a_ (0.67)
	2.41 _ab_ (0.48)	2.51 (0.64)	2.58 _b_ (0.58)
	2.72 _b_ (0.53)	2.38 (0.63)	2.49 _ab_ (0.64)
	3.31	1.85	7.73
	0.03	0.01	0.06
	0.038	0.159	<0.001

**Note:** Standard deviations appear in parentheses. Means within cells with different subscripts differ significantly at *p* < 0.05. *df* = 2255. WRV—workplace romance is valuable. DPWR—demand privacy about workplace romance. AWR—anti-workplace romance.

## Data Availability

Data are available from the author upon reasonable request.
